# The adaptation model of immunity: Is the goal of central tolerance to eliminate defective T cells or self‐reactive T cells?

**DOI:** 10.1111/sji.13209

**Published:** 2022-08-10

**Authors:** Masoud H. Manjili

**Affiliations:** ^1^ Department of Microbiology & Immunology, VCU School of Medicine VCU Massey Cancer Center Richmond Virginia USA

**Keywords:** anergy, adaptation, danger, self‐nonself, suppression, T cells, thymus, tissues, tolerance

## Abstract

The self‐non‐self model and the danger model are designed to understand how an immune response is induced. These models are not meant to predict if an immune response may succeed or fail in destroying/controlling its target. However, these immunological models rely on either self‐antigens or self‐dendritic cells for understanding of central tolerance, which have been discussed by Fuchs and Matzinger in response to Al‐Yassin. In an attempt to address some questions that these models are facing when it comes to understanding central tolerance, I propose that the goal of negative selection in the thymus is to eliminate defective T cells but not self‐reactive T cells. Therefore, any escape from negative selection could increase lymphopenia because of the depletion of defective naïve T cells outside the thymus, as seen in the elderly.

## 1 CENTRAL TOLERANCE: SNS MODEL VERSUS DANGER MODEL

In a discussion forum in response to Al‐Yassin,^1^ Fuchs and Matzinger^2^ proposed that, according to the danger model, the goal of central tolerance or negative selection in the thymus is not to induce self‐tolerance, but to induce tolerance to self‐dendritic cells.^2^ In fact, T cells that react with self‐dendritic cells expressing endogenously processed antigens are deleted during negative selection. Al‐Yassin proposes that administration of MHC‐incompatible cells prior to 18 days of gestation leading to tolerance is likely primarily due to central rather than peripheral tolerance since the expression of TcR genes does not begin until day 17 of gestation, and immunocompetent T cells appear after day 19 of gestation.^1^ However, Fuchs and Matzinger argue that the SNS model did not “reliably” induce tolerance in foetuses before the 18th day of gestation; in fact, the tolerance was induced only in less than one‐half of animals.^3^ According to Fuchs and Matzinger, tolerance versus immunization is dependent on the inoculum, not on the age of the recipient.^2^ They back up their hypothesis by the observations that neonatal female mice between 1 and 24 days of age become tolerant to the male‐specific H‐Y antigen, by an injection of male spleen cells, whereas they became immunized to H‐Y by an injection of male dendritic cells.^3^ They went on to argue that there is no need for thymic presentation of all peripheral tissue‐specific self‐antigens since peripheral tissues can induce tolerance to themselves due to a lack of costimulatory signals. According to the danger model, this is the mechanism of tolerance to bodily changes during puberty, pregnancy or carcinogenesis. There are several versions of the SNS model, which have been thoroughly reviewed by Matzinger;^4^ nevertheless, the backbone of all versions is the definition of self‐antigen (signal I) and the pattern recognition receptors specific for infectious non‐self (signal II).^5^, ^6^ When it comes to central tolerance, all versions of the SNS model appear to be focused primarily on the affinity of signal I or “self‐antigens” for TcR expressed by immature T cells.^7^ In addition, both the SNS and danger models agree that negative selection during central tolerance acts on immature lymphocytes while peripheral tolerance involves mature lymphocytes. The difference is related to the nature of tolerance being towards self‐antigens (SNS) or against self‐dendritic cells (Danger).

To address the question of why activated T cells should attack target cells outside the thymus but commit suicide in the thymus, both models propose that T cells are immature during negative selection; thus, they commit suicide upon activation. The danger model expanded on this question vaguely by suggesting that “immature thymocytes must go through a stage where they cannot receive costimulatory signals, and/or in which those signals are linked to cell death rather than cell activation. Thus, thymocytes specific for antigens presented by dendritic cells (both endogenous and captured) will become tolerant even if those dendritic cells have become activated. This is important, as the thymus is not a privileged site. It can, for example, be infected by viruses like LCMV. Thus, an infection in the thymus could lead to the activation and upregulation of co‐stimulatory signals on thymic dendritic cells”.^2^ The fact that dendritic cells in the thymus are mature because of the expression of co‐stimulatory molecules, such as CD40, B7‐1 and B7‐2 (signal II), regardless of infection,^8^ raises paradoxical questions such as how could thymic dendritic cells become mature in the absence of any infection or damage? Although LCMV infection hypothesis might explain some cases, this cannot explain maturity of APCs in the absence of infection or in animals housed under aseptic conditions. Also, expression of an endogenous danger receptor‐ligand in the thymus, if any, cannot be explained by the danger model as life‐threatening damage such as non‐physiological necrosis is unlikely present in the thymus in the absence of any dangerous/alarming signal. According to the danger model, “pathological and harmful death, like necrosis, exposes signals that activate DCs, whereas normal apoptotic cell death does not”^9^…“all organisms on our planet use DAMPs to signal that cell stress and tissue injury have occurred…(also) dysregulated emission of DAMPs plays a critical role in the induction of pathologies and diseases”.^10^ Such non‐physiological dangerous events do not seem to be present in the thymus, yet, mature DCs are present in the medulla. In fact, there is efficient antigen presentation by mature DCs and B cells in the thymus. To this end, the thymic stromal lymphopoietin (TSLP) induces maturation of thymic DCs expressing signals I/II in the medulla in the absence of any danger or infection.^11^ In addition, human B cells displaying elevated levels of MHC‐II and co‐stimulatory molecules B7.1 and B7.2 are present in the thymus.^12^ Nevertheless, low levels of CD28 expression in thymic T cells^13^ may only support T cell survival through the expression of Bcl‐xL without T cell activation by mature DCs or B cells.^14^, ^15^ In addition, the two‐step two‐signal process of T cell activation,^16^ which is required for activation of primed T cells in the periphery, may be absent in the thymus resulting in T cell priming by DCs or B cells without T cell activation. According to this model proposed by Peter Bretscher^16^ to correlate central and peripheral tolerance, the first step includes priming of naïve T cells by mature DCs presenting a nominal antigen, and the second step includes activation of the primed T cells by mature B cells expressing the same nominal antigen and delivering signal II for step 2. These mature B cells are generated via interaction with effector T cells specific for the same nominal antigen, which are not present in the thymus.

The second paradox is how could T cells that survive negative selection remain naïve despite interacting with activated dendritic cells? Even if these T cells might not have encountered their antigenic ligand in the thymus, such naïve T cells, according to the danger model, should be activated by self‐DCs in the periphery. After all, there is no direct evidence demonstrating that deleted T cells in the thymus are immature to undergo apoptosis upon activation during negative selection. Some indirect evidence are based on using thymocytes rather than recent thymic emigrants (RTE) T cells showing activation‐induced apoptosis mainly in double positive thymocytes,^17^, ^18^, ^19^ which have been misinterpreted as medullary thymic T cells being immature to undergo apoptosis upon activation.^20^ When RTE were used, T cells did not undergo apoptosis upon stimulation, accordingly, the authors argued against post‐thymic T cells requiring major maturation steps after leaving the thymus.^21^ In fact, mature T cells have been detected in the thymus by demonstrating that RANK‐ligand (RANKL) and CD40‐ligand, which are predominantly expressed in mature CD4 T cells, are involved in Aire expression in the thymus.^22^, ^23^ T cells that are ready to egress the thymus upregulate the expression of Kruppel‐like factor 2 (KLF2), a transcription factor for the naïve T cell markers including sphingosine‐1‐phosphate receptor‐1 (S1P1) and CD62L.^24^ While these observations support the maturity of medullary T cells, the assumption that they are immature is not supported by direct evidence. Such an assumption is made to justify that presumptive deletion of autoreactive T cells is because of their immaturity. The danger model differs from the SNS model in predicting what would happen if T cells escaped from negative selection. While the SNS model predicts that autoimmune diseases could occur, the danger model suggests that target tissues could induce tolerance. Prediction of autoimmunity by the SNS model is mainly based on the studies on *AIRE* deficiency or mutation associated with multi‐organ autoimmunity.^25^, ^26^ However, such multi‐organ autoimmunity may not necessarily be due to a failure to delete self‐reactive T cells that are not exposed to the *AIRE*‐coding self‐proteins, because only 4000 genes in the medullary thymic epithelial cells (mTECs) are expressed by *AIRE* ^27^, ^28^ out of more than 18 000 genes expressed independent from *AIRE* and represent 85% of the protein‐coding genome.^29^ Furthermore, the expression of *AIRE* is not restricted to the thymus; it is also expressed in circulating lymphocytes,^30^ testis,^31^ lymph nodes,^32^ liver and brain,^26^ suggesting that its only function may not be participating in central tolerance. In fact, *AIRE* is a pleiotropic gene controlling not only the expression of peripheral tissue antigens in the thymus but also interacting with other proteins and signalling pathways such that *AIRE* mutation in patients with autoimmune polyendocrinopathy‐candidiasis‐ectodermal dystrophy (APECED) syndrome inhibits TNF‐α production by inhibiting Declin‐1 signalling.^33^ Therefore, observations in *AIRE*‐deficient mice should not be fully attributed to the thymic negative selection. Some other studies observed GVHD‐like systemic autoimmunity in the absence of MHC class II and attributed that to impaired negative selection in spite of the observations that thymectomy did not cause autoimmunity.^34^ Also, negative selection of human T cells have been studied using non‐physiological conditions such as enforced expression of antigen or superantigen, showing no sign of autoimmunity.^35^, ^36^ Very recently, negative selection of human T cells were studied in a humanized mouse model in which human T cells recognized naturally expressed tissue‐restricted antigen in the human thymus and resulted in an impaired negative selection without autoimmunity.^37^ There are other observations challenging the notion that the goal of negative selection is the deletion of autoreactive T cells. For instance, in patients with sepsis, infection‐induced cytokine storm result in thymic atrophy and impaired negative selection without any autoimmunity. In fact, the dysregulation of negative selection due to thymic atrophy resulted in lymphopenia.^38^, ^39^ Interestingly, sepsis impedes the development of autoimmunity,^40^ perhaps because of the presence of defective/impaired T cells that escaped negative selection. Also, age‐related thymic atrophy that alters central tolerance, would not cause autoimmunity; rather, it results in an increased number of defective naïve T cells with a decline in TcR repertoire diversity.^41^ Similarly, studies on the medullary negative selection in a mouse model of OT‐1 CD8^+^ T cells revealed that T cells that escaped from negative selection did not cause autoimmunity, rather, such autoreactive T cells were functionally impaired.^42^ Autoreactive T cells are perhaps those that survived the medullary negative selection upon co‐stimulation by dendritic cells; and these are the ones that could cause autoimmunity.

Another challenge that the danger model, along with the SNS model, is facing is to explain CD34^+^ humanized NSG mice harbouring human T cells and mouse dendritic cells without compromising the central tolerance. These mice are exclusively humanized with CD34^+^, CD3‐depleted stem cells.^43^, ^44^ The CD34^+^ humanized mice differ from immune‐compromised mice reconstituted with human PBMCs in that central tolerance for human T cells towards mouse self‐antigens and self‐DCs takes place in the former but not the latter. In these mice, hypervariability of TcR^45^ allows flexibility of human T cells in recognizing mouse MHC‐peptide complex during positive selection. However, human T cells in these mice are not exposed to human MHC‐peptide complex or human dendritic cells, yet they tolerate human tumour cell lines carrying HLA mismatch.^43^, ^46^, ^47^, ^48^ Although no acute GVHD is evident in these mice, chronic GVHD is reported after 24 weeks in two NSG cohorts humanized with CD34^+^ grafts from different donors.^49^ According to the SNS model, human T cells should reject HLA mismatched tumour cells, which is not the case. According to the danger model, there should not be any chronic GVHD in these mice because GVHD causing human T cells while recognizing signal I, they cannot recognize a mismatch signal II to become tolerant in the thymus or become activated in the periphery by mouse DCs. To this end, costimulatory signals by mouse DCs should not be able to engage with CD28 expressed on human T cells during negative selection or during activation, even if major HLA becomes minor HA when taken up by mouse APCs. Nevertheless, chronic GVHD is reported in these mice. Regarding other immunocompromised mice, GVHD is more evident than that in CD34^+^ humanized mice despite the tolerance of human tumour cells. Again, GVHD cannot be explained by the danger model (DCs being of mouse origin), and allogeneic tumour tolerance cannot be explained by the SNS model.

## 2 CENTRAL TOLERANCE: THE ADAPTATION MODEL

Unlike the SNS model and the danger model, the adaptation model proposes that the purpose of negative selection is not to eliminate T cells reacting to self‐antigens or self‐APCs, rather, it is to select functional T cells that can survive the stress of signal I and eliminate defective T cells that are incapable of mounting survival signals upon receiving signals I/II. There are some observations suggesting that autoreactive T cells neither are deleted nor cause autoimmunity. For instance, the frequencies of the human CD8^+^ T cells specific for self and non‐self antigens are similar.^50^ Also, the Y chromosome‐encoded SMCY antigen‐specific T cells are present in both males and females with only a 3‐fold reduction in males.^50^ In the early mouse studies, the thymic negative selection was perceived as the deletion of autoreactive T cell because: (i) conclusions from the deletion of double positive T cells are generalized to the deletion of single positive T cells, (ii) key contributions of defects in anti‐apoptotic Bcl2/Bcl‐xL as well as elevated levels of pro‐apoptotic Bim during negative selection of double positive and single positive T cells, respectively, are overlooked, and (iii) TcR transgenes used were almost always originated from T cell clones that were the best responders to a given antigen, which might not naturally exist in wild‐type mice. In comparison to wild‐type mice, the introduction of TcR transgenes results in an artificial overexpression of the TcRs as well as the early expression of mature TcR at the double‐negative (DN) stage.^51^ This could reduce the efficiency of positive selection and affect repertoire skewing in transgenic mice. Such reduced efficiency in positive selection has been reported for TcR transgenes when they exceed 5%.^52^ Therefore, “deletion of autoreactive T cells” is an incomplete understanding of the thymic negative selection. The adaptation model proposes that “deletion of defective T cells” is the main purpose of negative selection. Deletion of such defective T cells during negative selection of double positive T cells in the cortex has been reported to involve the engagement of TcR and the costimulatory molecule CD28 to induce apoptosis of CD4^+^CD8^+^ T cells.^53^, ^54^ It is well‐established that priming of T cells through antigen recognition and co‐stimulation results in PKC signalling and ER stress which could kill T cells if they fail to mount ER‐stress response through upregulation of the expression of the ER chaperone GRP78^55^ or elevation of anti‐apoptotic Bcl‐xL.^56^, ^57^ In fact, T cells that mount survival signals through CD28 co‐stimulation to survive the negative selection in the cortex.^58^ Such inability of T cells in mounting survival signals could also be due to higher expression of pro‐apoptotic proteins during antigen recognition and co‐stimulation. This is the case during negative selection of single positive T cells in the medulla where the pro‐apoptotic Bim is actively involved in negative selection.^42^ Interestingly, escaping from such negative selection in the medulla did not lead to autoimmunity, rather, such autoreactive T cells were functionally impaired.^42^ Therefore, any escape from negative selection would result in the presence of circulating T cells with impaired function and homeostasis or massive apoptosis of defective T cells upon activation in the periphery rather than causing autoimmunity.^42^, ^59^, ^60^ This is the case in patients with sepsis where thymic atrophy could lead to the escape of defective T cells that undergo apoptosis during antigen recognition or co‐stimulation in the periphery leading to lymphopenia.^38^, ^39^ Also in the elderly, age‐related thymic atrophy that alters central tolerance, would not cause autoimmunity; rather, it results in a decline in naïve T cells,^61^ an increased number of defective naïve T cells with a decline in TcR repertoire diversity,^41^ as well as defective T cells that are inefficient in priming.^62^ Also, lymphopenia increases with age because of the reduction of naïve T cells;^63^ perhaps, impaired T cells emerging from inefficient central tolerance are defective in mounting survival pathways in circulating T cells. With regard to antigenic affinity, deletion of T cells during negative selection is not limited to the deletion of T cells with high affinity TcR for self‐antigens or self‐APCs. It has been reported that *Aire*‐deficient and wild‐type mice show no differences in the TcR Vβ repertoire,^64^ neither was there any major autoimmunity in *Aire*‐deficient mice except for a mild autoimmune‐like dry eyes.^64^ In addition, transgenic mice expressing HA or ova by mTECs under the control of *Aire* where DCs also presented both HA and ova at detectable levels, the antigen‐specific CD4^+^ thymocytes were not deleted.^65^ Also, deletion of the ova‐specific OT‐I CD8^+^ thymocytes did not require MHC‐I expression by bone marrow‐derived APCs.^66^ It is not the affinity of thymocytes for self‐antigens determining negative selection as thymocytes that receive strong TcR‐signalling could differentiate into Treg cells.^67^ In addition, thymic emigration decreases in *Aire* ^*−/−*^ mice,^68^ suggesting that autoreactivity is not because of the escape of otherwise deleted T cells and their addition to the pool of surviving T cells. Therefore, T cell deletion is not because of the activation but it could be because of T cells' inability to mount pro‐survival Bcl2/Bcl‐xL upon receiving signal I during homeostasis or signal I and a weak costimulatory signal II from dendritic cells during priming.^14^ Thymic T cells express low levels of CD28^13^ which may only support T cell survival through the expression of Bcl‐xL without T cell activation by mature DCs or B cells.^14^, ^15^ It has been reported that overexpression of the anti‐apoptotic protein Bcl‐2 can inhibit negative selection under certain conditions^69^ as can the loss of the pro‐apoptotic family members BIM^70^ or Bcl‐2 antagonist/killer (BAK) and Bcl‐2‐associated X protein (BAX).^71^ Also, when mixed bone marrow chimeras were created with cells derived from both CD28‐deficient and wild‐type mice, the CD28^+^ T cells had a selective advantage over the CD28‐deficient T cells.^72^

According to the adaptation model of immunity, survival signals regulating the thymic negative selection are orchestrated via the expression of adaptation receptors (AdRs) on T cells and their nominal adaptation ligands (AdLs) on the thymic APCs.^59^, ^60^ The CD28/B7 co‐stimulation is an example of the AdR/AdL pathway, but it is not the only one, some of which is yet to be discovered. The endothelin receptor A (ET_A_) could be another AdR which upon binding to its ligand ET‐1 induces cell survival by mounting Bcl‐xL.^73^ In the thymus, activation of ET_A_ support survival of thymocytes.^74^ B7‐H1 (PD‐L1) is another bi‐directional receptor acting as a ligand to induce anergy in PD‐1‐positive T cells, and acting as an AdR to induce anti‐apoptotic genes in B7‐H1‐positive target cells.^75^ Because of ignoring the anti‐apoptotic function of B7‐H1 and focusing on its function on PD‐1^+^ T cells, this bi‐directional receptor has been mischaracterized in immunology literature as a ligand, PD‐L1. Constitutive expression of B7‐H1 in the immune privileged sites such as cornea and retina protects them from rejection following corneal allograft, despite infiltration of CD4^++^ T cells; however, blockade of B7‐H1 accelerates allograft rejection.^76^ These observations suggest that expression of B7‐H1 on target cells induces survival signal by PD‐1^+^ effector T cells. Similarly, the expression of B7‐H1 on activated T cells supports their survival such that B7‐H1‐deficient T cells express lower Bcl‐xL, which is an anti‐apoptotic gene, than wild‐type cells and are more sensitive to apoptosis in vivo.^77^ Defects in the expression of AdRs by T cells or AdLs by APCs could result in AICD in T cells during activation. For instance, hepatic APCs induce apoptosis in T cells during activation, whereas splenic APCs support survival of activated T cells.^78^ The AdR/AdL pathway is not limited to membrane receptor‐ligand interactions, they could also include intracellular pathways. For instance, the ER stress induced upon signal I could lead to T cell apoptosis. To this end, the KDEL receptor‐mediated pathway rescues naïve T cells from apoptosis upon priming.^79^ It was reported that inefficient KDEL1 gene function results in enhanced apoptosis of naïve T cells during stress response.^79^ The KDEL1 interacts with the ER‐resident chaperons to mount T cell survival.^80^ Corticoid receptors are another example of AdRs supporting thymocyte survival. The Nur77 and Helios transcription factors are upregulated in thymocytes upon the TcR/CD28 stimulation, inducing negative selection. Interestingly, glucocorticoids inhibit upregulation of Helios and Nur77 in negatively selected TcR‐stimulated mouse thymocytes.^81^ Also, self‐reactive T cells in the thymus are not immature, as suggested by the SNS model, rather they have matured into CD4^+^ or CD8^+^ T cells from immature CD4^+^CD8^+^ T cells during positive selection. The maturation markers RANK ligand (RANKL) and CD40 ligand are detected on thymic T cells.^22^, ^23^ Also, T cells that are ready to egress the thymus upregulate the expression of Kruppel‐like factor 2 (KLF2), a transcription factor for the naïve T cell markers including sphingosine‐1‐phosphate receptor‐1 (S1P1) and CD62L.^24^

In conclusion, it is likely that T cells expressing anti‐apoptotic Bcl‐2/Mcl‐1 during co‐stimulation in the thymus, will survive negative selection and those that fail to express these molecules will be deleted because of the activation of pro‐apoptotic Nur77/Bim/Bax/Bak molecules.^82^ Similarly, positive selection depends on the expression of anti‐apoptotic Bcl‐xL in T cells; otherwise, pro‐apoptotic Bim/Bax/Bak would delete T cells in the thymic cortex. Therefore, tolerance of mismatched or dangerous human tumour cell lines in humanized CD34^+^ NSG mice is because the outcome of the immune response is not determined by signal I or II, rather, it is determined by signal III, which is the expression of the AdRs on target cells engaging the AdLs on T cells to relay survival signals in target cells, as previously described by the adaptation model of immunity.^59^, ^60^ Therefore, regardless of HLA mismatch or alarming signals, human tumour cell lines could receive survival signals from human T cells and be tolerated in these mice.

## Data Availability

Data sharing not applicable to this article as no datasets were generated or analysed during the current study
